# Breakthrough Pain: The Importance of Baseline Analgesic Regimen with Opioids

**Published:** 2012-04-30

**Authors:** Maddalena Zampi, Antonietta Morabito, Fabiana Salvato, Annamaria Vinciguerra

**Affiliations:** 1 Department of Surgery, Anaesthesiology and Emergency-Resuscitation “Giuseppe Zannini”, University of Naples “Federico II”, Italy

**Keywords:** Breakthrough Pain, μ-receptor, Polimorphisms

## Abstract

Pain is one of the most common and often most feared symptoms in patients with cancer. Ongoing or progressive pain is physically debilitating and has a marked impact on quality of life. During their illness, at least 70% of patients will experience pain sufficiently severe to require chronic opioid treatment. Moreover, Breakthrough Pain (BTP) consists in transitory exacerbations of pain that occurs on a background of otherwise stable pain in a patient receiving chronic opioid therapy. An inadequate baseline therapy with opioids can be one of the causes of BTP.

We will examine the molecular issues that influence the response of patients to opioids. Finally, we will discuss about the importance of individualizing therapy.

## I. INTRODUCTION

Cancer-induced pain is usually described under 3 headings: acute pain, chronic pain and Breakthrough Pain (BTP). Acute pain, for example, may result from the pathologic fractures that occur when tumors invade bone. One form of chronic pain may occur when cancer directly invades nerves causing severe neuropathic pain [1,2].

BTP consists in transitory exacerbations of pain that occurs on a background of stable pain controlled by baseline opioid regimen. BTP characteristically reaches peak intensity within a matter of minutes, requiring treatment with a rapidly acting, potent opioid.

Clinical definitions of BTP have been further refined by distinguishing between the following [3]:

- *incident, predictable pain*: a consistent temporal causal relationship with a predictable motor activity such as movement, defecation, urination or breathing;
- *incident, unpredictable pain*: an inconsistent temporal causal relationship with predictable motor activity;
- *idiopatic pain*: not associated with a known cause and usually of longer duration compared with incident pain;
- *procedural pain*: pain related to a therapeutic intervention such as dressing a wound;

Some authors in the past also described the *end-of-dose BTP* as pain occurring before a scheduled dose of an around-the-clock analgesic. However, it is not properly a subtype of BTP, because this pain is frequently related to underdose of the opioids used for baseline therapy and it more often occurs during titration phase.

With respect to analgesics, the World Health Organization (WHO) has devoloped a 3-step “analgesic ladder” to guide management of cancer pain [4], based on the pain’s severity, estimated by use of a 1 to 10 numeric rating scale [Table I].

For treatment of BTP, there is a need for rapidly acting, powerful “rescue” analgesic. Intravenous morphine has been used for this purpose with reported success. However, fentanyl seems to be preferable for treatment of BTP. Because of its low molecular weight and lipid solubility, fentanyl is well suited for delivery via transmucosal or intranasal system. When fentanyl is administered through buccal mucosa is rapid, providing significant pain relief within 15–30 minutes [5]; on the other hand, intranasal fentanyl spray can produce a substantial reduction of BTP as soon as 15 minutes after it is taken [6].

Frequent exacerbations of pain intensity could be the sign of inadequate baseline analgesic therapy (beyond disease’s progression). Moreover it has been demonstrated that there are individual variations in morphine absorption or alterations in its volume of distribution; these findings could explain the relationship between the variation of opioid’s plasmatic concentration during the day, which depends on the individual, and the analgesic activity.

In addition to this, the physicians underline the great variability in patient’s response to opioids and the need to individualize opioids’ use: patients differ in drug’s sensibility (some of them need higher doses to gain the same outcome) and show different responses to different opioids.

Finally, we know that cross-tolerance among opioids is incomplete and the conversion from an opioid to a different one can be difficult, because of the limits of equianalgesic tables[7].

Concerning the molecular aspects, there are many issues that influence patient’s response to opioid therapy: the receptor (polymorphism of the gene that codes receptor μ and beta-arrestine), the translation of intracellular signaling (role of regulator of G protein signaling protein family), the passage across the blood–brain barrier (G-protein polymorphisms) and the metabolism (cytocrome P450) [8].

## II. POLIMORPHISM OF MU OPIOID RECEPTORS

Opioid receptors are widely distributed in central and peripheral nervous system.

Three opioid receptors have been identified: μ, δ and κ. Morphine and other commonly used opioids, including oxycodone, hydromorphone, methadone and fentanyl, act primarily on the same target receptor, the μ-opioid receptor (MOR). In addition, oxycodone, hydromorphone and buprenorphine may have clinically important activity on other opioid receptors. The structure of the “μ opioid analgesics” ranges from minor variations of the morphine alkaloid structure, such as codeine, to markedly different structures, such as fentanyl and methadone [9].

These analgesics share the same general pharmacological profile as morphine, including analgesia, inhibition of gastrointestinal motility, and respiratory depression. However, differences in the clinical pharmacology of these μ receptor agonists seriously questioned: how might drugs that act on the same receptor differ so markedly?

Nociceptive threshold varies depending on genic expression, as it was observed in animals lacking μ-receptor (homozygotes), heterozygotes and carriers of the normal receptor. However, some allelic variants can modify the response to opioids maybe altering transcriptional factors. Cytokines, in particular, are able to regulate some transcriptional factors and influence the activity of μ-receptor.

The receptor μ has been cloned 15 years ago and it was named MOR-1; it is made of 4 exons. The physicians found out that giving antisense (a sort of an antibody which inactivates a part of the receptor) for exon 1, they obtained a reduction of the analgesic action of morphine but not of its active metabolite, morphine-6β-glucuronide (M6G). This event didn’t happen with antisense for exon 2 and 3.

Many variants of MOR have been discovered. For example, MOR-1A lacks of exon 4 which is partially transcribed on exon 3; in MOR-1B exon 4 is replaced by exon 5. Other variants differ for the intracellular terminus. It has been observed that, although the variants have similar affinity for morphine, they show differences in activating the receptor, that means a different efficacy [10].

Knockout methodology has been another useful tool. In this technique the gene is definitively deleted, while in the antisense technique mRNA is temporary inactivated. For example, the lack of the gene for MOR-1 prevents the action of morphine while other opioids retain the analgesic effect even though it is reduced.

The hypothesis that retain of the analgesic effect depends on the link with other opioid’s receptors (such as δ and κ), has been rejected by the observation that residual analgesia was erased by MOR-selective antagonists. The pharmacological profile of M6G is similar to that of morphine and its analgesic effects are reversed by naloxonazine, a MOR-antagonist. However, the actions of M6G can be differentiated from morphine in several ways. Firstly, the antagonist 3-O-methylnaltrexone (3-methoxynaltrexone) selectively blocks M6G-induced analgesia at doses that are ineffective against morphine. Secondly, M6G and several other drugs that act on the MOR retain their analgesic activity in CXBK mice, which are insensitive to morphine [11].

Genetic polymorphisms can also involve a single nucleotide (Single Nucleotide Polymorphism, SNP) in the DNA sequence of MOR’s gene (OPRM1). The most known mutation in this receptor is 118A > G SNP, a variant of 10–15% of caucasian people, where asparagine is replaced with aspartate on exon 1; this variation causes the loss of the glycosilation site on the extracellular terminus of MOR. This polymorphism is associated with a reduction of effects, above all of the side effects, of M6G, alfentanil and morphine.

Homozygotes, unlike heterozygotes and normal genotypes, require higher doses of opioids to gain the same analgesic outcome. Moreover, patients with 118GC genotype require higher doses of morphine for postoperative pain than patients with 118AG or AA genotype. Recently, the 118A>G genotype was associated with the level of pain control: homozygotes 118A showed a reduction in pain intensity greater than homozygotes 118G and heterozygotes. However, these data are belittled by the observation that patients not responsive to morphine didn’t have a different genotype from responsive patients.

Moreover, also side effects could have a genetic explanation. For example, in volunteers nausea, vomit and respiratory depression were associated to 118A>G polymorphism and this polymorphism seemed to cause a general reduction of clinical effects of opioids and higher analgesic request [12].

Polymorphisms involved in opioid response are complex and multigenic, with a great number of alleles. Furthermore, some genetic variations interfere with the affinity between receptor and opioid and lead to a different activation e/o desensitization.

For example, they can influence the transcription of beta-arrestine, an intracellular protein involved in the process of receptorial desensitization and internalization. It was observed a genetic polymorphism for beta-arrestine in patients showing a phenotype with low analgesic response to morphine or prevalence of side effects morphine-induced [13].

## III. REGULATOR OF G-PROTEIN SIGNALING (RGS)

MOR belongs to the G protein coupled receptor family, with an extracellular N terminus, an intracellular C terminus, and seven membrane-spanning domains in between that comprise the binding pocket for the drug.

Heterotrimeric G proteins comprise three proteins: one Gα subunit and a heterodimer of β and γ subunits. MOR interacts with the Gα_i/o_ class of adenylate cyclase inhibitory Gα proteins [14], that is comprised of Gα_i_ and Gα_o_. In the resting state the Gα subunit is bound to the guanine nucleotide GDP and is in complex with the β and γ subunits. Activation of MOR by agonists leads to dissociation of GDP from the Gα subunit, which is replaced by GTP, and separation of the Gα-GTP from the βγ heterodimer. The now active Gα-GTP and βγ subunit complex interact with intracellular signaling proteins, including inwardly rectifying potassium channels, calcium channels, phospholipase C and the mitogen-activated protein kinase (MAPK) pathway as well as a variety of adenylate cyclase isoforms, to generate physiological responses. The intracellular signal is terminated by endogenous GTPase activity of the Gα subunit which hydrolyzes the Gα bound GTP to GDP. Gα-GDP can no longer activate effector proteins and moreover re-associates with the βγ heterodimer to terminate βγ signaling and reform the GDP-bound heterotrimer. In this way, heterotrimeric G protein substrate can be recycled for a subsequent round of receptor activation. The enzymatic GTPase activity of the Gα_i/o_ subunits is slow with a GTP turnover rate of 2–5 per minute. This is not sufficiently rapid to turn off the signal and allow responses to subsequent incoming signals. However, the rate of hydrolysis of GTP is accelerated approximately 100-fold by the binding of a regulator of G protein signaling (RGS) protein to the active GTP-bound Gα subunit. RGS proteins thus function as GTPase accelerating proteins (GAPs) and, by rapidly removing the active Gα-GTP and βγ species, act as negative regulators of G protein-coupled receptor (GPCR) signaling. Since RGS proteins negatively regulate GPCR signaling, these accessory proteins have been implicated in the actions of opioids [15].

There are more than 30 mammalian RGS proteins, defined by the presence of a RGS homology (RH) domain, a region of 125 aminoacids that binds the Gα-GTP subunit and accelerates GTP hydrolysis. RGS proteins are divided into several families based on the RH domain homology and on the existence of a variety of additional structural domains. Consequently, it is difficult to identify the individual RGS protein or proteins that might be specifically responsible for the negative modulation of opioid signaling in a given tissue and therefore relevant to a particular physiological response. Certainly, there are so many RGS proteins that redundancy is likely. For example, RGS2, RGS4, RGS6, RGS7, RGS8, RGS9-2, RGS11, RGS19 and RGS20 have all been reported to act as GAPs for MOR signaling in different systems. Therefore, it is difficult to ascertain if an observed lack of physiological effect in response to knockdown or knockout of a single RGS protein is meaningful.

There are currently no useful pharmacological inhibitors of RGS proteins available with which to probe roles for these proteins, although progress is being made in this area [17].

## IV. P-GLYCOPROTEIN

The membrane-bound drug transporter P-glycoprotein influences drug absorption and drug excretion. It regulates the transfer of opioids across the blood–brain barrier and can actively pump opioids out of the central nervous system (CNS). P-glycoprotein knockout mice, which are completely devoid of P-glycoprotein activity, have enhanced absorption and high CNS concentrations of P-glycoprotein substrates (e.g. morphine, fentanyl, and methadone) with associated prolongation of analgesia. Administration of cyclosporin, a P-glycoprotein inhibitor, results in increased fentanyl and morphine-induced analgesia. Interindividual variability in P-glycoprotein activity is well recognized and genetic variations in the multidrug resistance gene (MDR-1), which encodes for P-glycoprotein, have been associated with resultant alterations in P-glycoprotein activity [18].

Since P-glycoprotein modulation of opioid CNS levels varies substantially among different opioids, the effects of genetic variation altering P-glycoprotein activity are different, depending on the opioid in question. MDR-1 gene is therefore a good candidate gene for influencing the analgesic response to opioids and the prevalence of opioid-induced CNS side effects. Whereas SNPs have been identified in the MDR-1 gene: two mutations (C3435T and G2677T/A) have been associated with differences in P-glycoprotein expression or function. A variation in the G2677T/A genotype has also been shown to alter drug levels and drug-induced side effects [19].

## V. P450- CYTOCROME

As for the genetic variations of opioid receptors, also the enzymes involved in drugs’ metabolism can influence the response to opioids. All opioids are metabolized mainly by P450-Cytocrome and for a lesser extent by UDP-glucuronosiltransferase (UGT).

Although CYP3A4 is involved in the metabolism of many opioids, CYP2D6 shows a greater genetic instability and it is involved in generating strong metabolites with a greater affinity for μ-receptor. On the other hand, functional effects of genetic variants of UGT have not been defined yet.

The CYP2D6 gene is highly polymorphic, with approximately one hundred of identified allelic variants; among these variants some have a deleted function, some have a reduced function, some others are duplicated and so they have an increased function. The main substrates of this enzyme are: benzodiazepines, haloperidol, carbamazepine, fentanyl, alfentanil and methadone, antifungal agents, many antibiotics, calcium antagonists, antihistamines, antiarrhythmics, steroids and immunosupressants. This wide range of drugs is the main reason of a great possibility for a competitive interaction, which depends on drugs’ concentration, affinity between enzyme and substrate, contemporary administration of drugs that induce the enzyme [20].

## VI. TOLERANCE

Side-effects and analgesic responses can vary significantly among patients. Although all MOR-agonists are associated with tolerance after repeated usage and exhibit cross tolerance to each other, the degree of cross tolerance varies widely. Indeed, we use incomplete cross tolerance to restore analgesic sensitivity in highly tolerant patients. When switching a patient who is highly tolerant to one MOR-selective opioid into another opioid, a process that is termed “opioid rotation”, analgesia is often restored by the second drug at doses ≥50% below the predicted equivalent dose determined by relative potency studies in naive subjects. Many issues could play a role in incomplete tolerance, including metabolism and pharmacokinetics and MOR multiplicity [21].

Moreover, incomplete cross tolerance can depend on differences in agonistic efficacy. The effect of an opioid depends on the number of filled receptors: the more they are filled, the less intrinsic activity the opioid has got. Therefore, if the receptorial reserve is great, the intrinsic efficacy of that opioid will be great.

For example, on the one hand morphine is an agonist with low intrinsic activity and has to fill many receptors, so it is associated with high degree of tolerance; on the other hand methadone needs to fill a lower number of receptors to obtain the same outcome. That’s the reason why the switching from an opioid which is loosing its analgesic properties to an opioid with greater efficacy, can help us to improve the therapy.

## VII. HYPERALGESIA

Many studies have demonstrated that opioids can unexpectedly produce an increased response to a painful stimulus (hyperalgesia) or a painful response to a harmless stimulus (allodynia). This phenomenon can develope in response to both chronic and acute exposure to opioids. In patients with chronic pain the administration of repeated doses of opioids gradually reduces the threshold of response to nociceptive stimuli. This status can lead the patient to a condition of subliminal withdrawal (because of the low plasmatic concentration among the doses) and to the increase of progressive neuronal discharge and the further reduction of nociceptive threshold [22]. The clinical consequence is the loss of analgesic efficacy. Therefore the reduction of analgesic efficacy can depend on many issues such as tolerance, hyperalgesia or progression of the disease.

Moreover, antinociceptive system is balanced by pronociceptive system (hypothesis of Celerier) [23]. So what happens when starting a therapy with opioids? The pronociceptive system gets upregulated and, in order to reach a new balance, the fisiologic excitatory response opposes to the inhibitory action of opioids. Clinically the patient will complain of hyperalgesia until a new balance in neuronal activity will be reached.

## VIII. CONCLUSION

Opiates play a basic role in the management of pain. The treatment of pain requires individualization of therapy because of the wide range of responses of patients to individual drugs [24].

Frequent events of BTP can result from inadequate daily therapy. The dosage of opioids will be considered satisfactory when the patient will have less than 2–3 events of BTP a day. A better management of the scheduled doses can further reduce the events of BTP.

There is no ceiling effect for analgesia and doses can be escalated until limiting side effects are reached, at which point patients can be switched to an alternative opioid. Incomplete cross-tolerance can explain the utility of opioid rotation. Most clinical analgesics act through μ receptors. Recent studies now indicate that there is a great number of splice variants of this receptor. Pharmacologic differences among these variants may help to explain differences in the actions of various opioids despite the fact that they all are MOR-selective. Differences in the generation of these variants may also help to explain the wide genetic variability of response among patients taking these drugs and may provide insights into why clinicians still need to individualize therapy for their patients.

## Figures and Tables

**Table I:** The WHO 3-step guide for treatment of pain in patients with cancer

Steps and pain intensity (scale: 1–10)	Recommended medications
Mild pain (1–4)	Acetaminophen, NSAIDS (± adjuvants)
Moderate pain (5–6)	Hydrocodone, oxycodone, tramadol (± non opioid analgesics) (± adjuvants)
Severe pain (7–10)	Hydromorphone, fentanyl, methadone, oxycodone (±nonopioid analgesics) (± adjuvants)
